# Supplementary material for: Cigarette Smoke and Decreased DNA Repair by Xeroderma Pigmentosum Group C Use a Double Hit Mechanism for Epithelial Cell Lung Carcinogenesis
Source: bioRxiv. 2025 Feb 27:2025.02.22.639660. Preprint. [Version 1] doi: 10.1101/2025.02.22.639660 (PMC11888293; doi:10.1101/2025.02.22.639660)
Supplement: Supplement 1 [file media-1.pdf]

Supplemental Figures and Methods

Supplemental Figures:

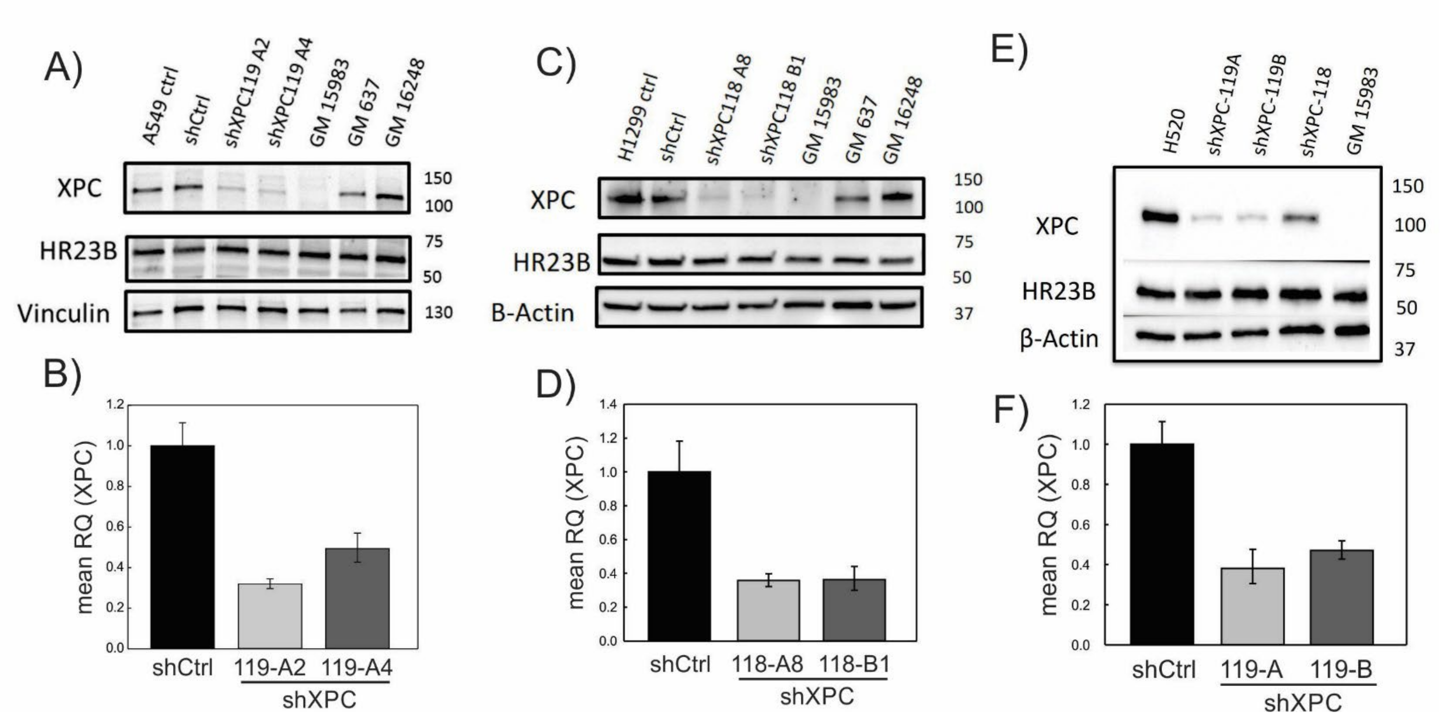

Supplemental Figure 1: Stable XPC knock-down cell line development. Lentiviral siRNA to XPC performed in 3 cancer cell lines: A549 (A,B), H1299 (C,D) and H520 (E, F). Western blot analysis shows decreased XPC protein (A, C, E) expression. Decreased *XPC* mRNA expression by RT-qPCR (B, D, F) expression. shCtrl (siRNA to non-targeted XPC expression levels are measured by Western blot analysis in A549 cells after stable KD of XPC by lentiviral shRNA and compared with loading control (β-actin), with specificity confirmed by expression levels of the XPC complexing protein, HR23B (A). Two XPC KD clones (A549: shXPC119-A2 and 119-A4 are shown with the parent cell line (A549), nontargeting shRNA control (shCtrl) and control fibroblast cell lines from an unaffected human (GM637), and XPC-deficient proband unmodified (GM15983) and reconstituted with XPC cDNA (GM16248). Calculated XPC expression levels normalized to loading control and compared with shCtrl are shown below a representative immunoblot. XPC RNA expression levels by qRT-PCR are shown compared with shCtrl (B). Likewise, (C) showed XPC expression levels as measured by Western blot analysis in H1299 cells as well as two XPC KD (shXPC 118 A8 and shXPC 118 B1). (D) shows the decreased XPC RNA expression normalized to shCtrl. Finally, (E) showed XPC expression levels as measured by Western blot analysis in H520 cells as well as two XPC KD (shXPC 119A and shXPC 119B) and (F) shows the decreased XPC RNA expression normalized to shCtrl. (mean ± SD; n = 3). MW = molecular weight.

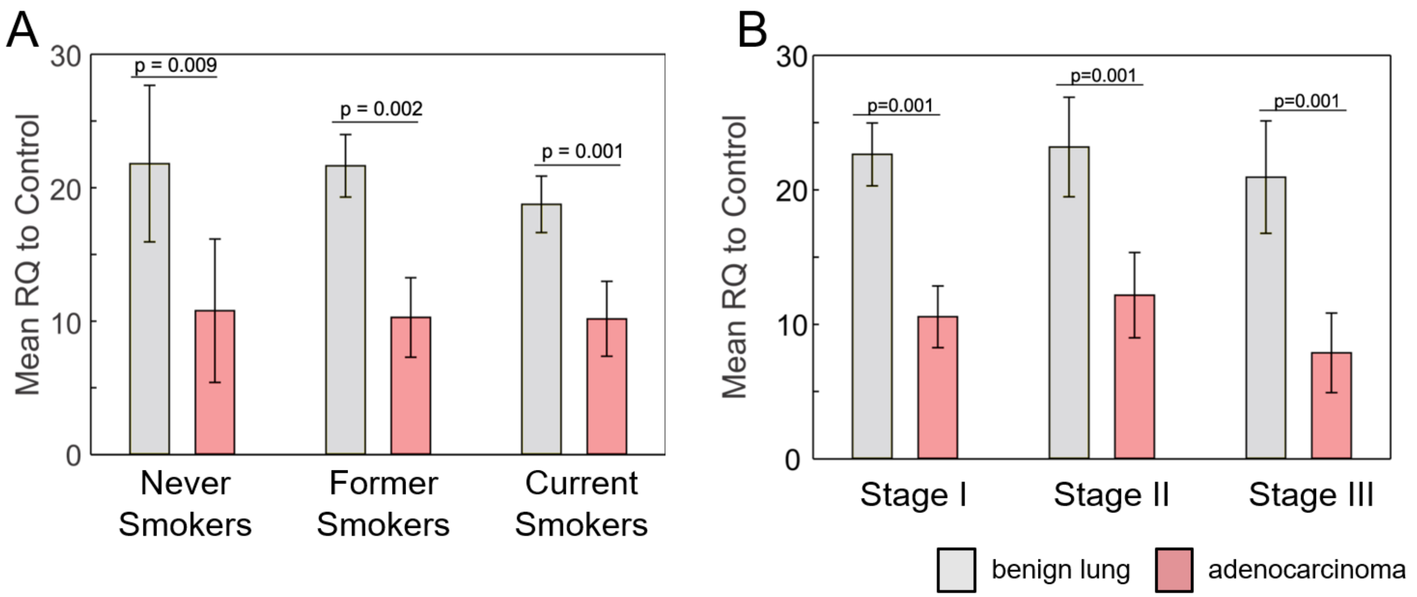

Supplemental Figure 2: Subgroup analysis of XPC mRNA expression divided by A) smoking status and B) stage.

Supplemental Tables:

Table 1: Human lung adenocarcinoma samples - Demographics

Demographics	Percent/Average (SD)
Number of subjects (matched specimens)	45 (44 used for analysis)*
Age	63.5 (+/- 10.8)
Male Sex	43%
Race	
White	82%
Black	16%
Asian	2%
Cigarette smoking status	
Ever Smoker	61.4%
Never Smoker	20.5%
Current Smoker	16%
Unknown/Not recorded	22.7%
Stage at time of tissue collection	
Stage I	41%
Stage II	39%
Stage III	14%
Stage IV	2%
Unknown/Contradictory	5%

*insufficient/poor quality RNA yield from tumor

Table 2: Human lung squamous cell carcinoma samples - Demographics

Demographics	Percent/Average (SD)
Number of subjects (matched specimens)	36
Age	63.9 (+/- 7.7)
Male Sex	56%
Race	
White	94%
Black	6%
Cigarette smoking status	
Ever Smoker	86.1%
Never Smoker	0%
Current Smoker	41.7%
Unknown/Not recorded	13.9%
Stage at time of tissue collection	
Stage I	33%
Stage II	42%
Stage III	17%
Stage IV	0%
Unknown/Contradictory	8%
